# Carrier Compensation Induced by Thermal Annealing in Al-Doped ZnO Films

**DOI:** 10.3390/ma10020141

**Published:** 2017-02-08

**Authors:** Takashi Koida, Tetsuya Kaneko, Hajime Shibata

**Affiliations:** 1 Research Center for Photovoltaics, National Institute of Advanced Industrial Science and Technology (AIST), Central 2, Umezono 1-1-1, Tsukuba, Ibaraki 305-8568, Japan; tetsuya-kaneko@tokai-u.jp (T.K.); h.shibata@aist.go.jp (H.S.); 2 School of Engineering, Tokai University, 4-1-1, Kitakaname, Hiratsuka, Kanagawa 259-1292, Japan

**Keywords:** transparent conducting oxides, ZnO, doping, carrier compensation, mobility

## Abstract

This study investigated carrier compensation induced by thermal annealing in sputtered ZnO:Al (Al_2_O_3_: 0.25, 0.5, 1.0, and 2.0 wt %) films. The films were post-annealed in a N_2_ atmosphere at low (1 × 10^−23^ atm) and high (1 × 10^−4^ atm) oxygen partial pressures (*P*_O2_). In ZnO:Al films with low Al contents (i.e., 0.25 wt %), the carrier density (*n*) began to decrease at annealing temperatures (*T*_a_) of 600 °C at low *P*_O2_. At higher *P*_O2_ and/or Al contents, *n* values began to decrease significantly at lower *T*_a_ (ca. 400 °C). In addition, Zn became desorbed from the films during heating in a high vacuum (i.e., <1 × 10^−^^7^ Pa). These results suggest the following: (i) Zn interstitials and Zn vacancies are created in the ZnO lattice during post-annealing treatments, thereby leading to carrier compensation by acceptor-type Zn vacancies; (ii) The compensation behavior is significantly enhanced for ZnO:Al films with high Al contents.

## 1. Introduction

Transparent conducting oxide (TCO) films have been widely used as window electrodes in optoelectronic devices such as flat panel displays and solar cells. Most TCO films are based on In_2_O_3_, SnO_2_, or ZnO, of which ZnO is the most important due to its advantages of low cost and nontoxicity compared to In_2_O_3_. Additionally, ZnO films exhibit higher conductivity than SnO_2_ films at relatively low growth temperatures. Recently, ZnO films have been used in thin-film solar cell applications [1], however, they typically exhibit low conductivity compared to In_2_O_3_-based TCO [2]. The most important properties of TCO films for use as window electrodes are their conductivity and absorption coefficient. Both of these parameters are related to the carrier density (*n*) and mobility (*μ*) [3]. The carrier density (*n* = *N*_d_ − *N*_a_) is determined by the donor (*N*_d_) and acceptor (*N*_a_) densities, and these two values influence *μ* through Coulomb interactions that cause free electron scattering by charged donor and acceptor defects. In the case of Al-doped ZnO (ZnO:Al) films, for example, the dominant donor is the singly charged dopant Al_Zn_•, whereas the compensating acceptor is considered to be a doubly charged zinc vacancy (v_Zn_″) [4,5,6,7]. Hence, suppressing v_Zn_″ is important for achieving both high *n* and *μ*.

The changes in concentration of intrinsic defects such as v_Zn_″ are typically described using Brouwer diagrams [8]. Lany et al. calculated intrinsic defect densities and *n* for pure ZnO and ZnO:Al as a function of growth temperature and oxygen partial pressure (*P*_O2_) based on the formation energies of the defects and the thermodynamics of the relevant defect reactions [6]. In Zn_0.99_Al_0.01_O, the donor density remains uncompensated at low *P*_O2_, whereas *n* decreases with increasing *P*_O2_ as a result of compensation by the v_Zn_″ acceptor. Zakutayev et al. have also investigated the connection between defect theory and thin-film growth of ZnO:Ga by comparing theoretically and experimentally obtained *n* values [9]. They demonstrated that the high conductivity of the films was due to the highly non-equilibrium, metastable state that resulted from growth by pulsed laser deposition (PLD) at relatively low temperatures and low *P*_O2_. In addition, Look et al. determined *N*_d_ and *N*_a_ densities from *μ* and *n* values for ZnO:Ga thin films grown by PLD based on a mobility analysis in which *μ* was mainly determined by the scattering from ionized donors and acceptors [10].

The purpose of the present work is to investigate the carrier compensation behavior in sputtered ZnO:Al films during post-annealing treatment at low (1 × 10^−23^ atm) and high *P*_O2_ (1 × 10^−4^ atm). Because the sputtering process is known to be a non-equilibrium method, the as-deposited ZnO:Al films are far from an equilibrium state. During post-annealing at high temperatures under constant *P*_O2_, the film equilibrates with O_2_ through interaction with the surrounding gas phase. Consequently, the density of v_Zn_″ defects should approach a certain value determined by the *P*_O2_ and annealing temperature. Therefore, monitoring the variation of *n* values can be used to evaluate carrier compensation induced by the thermal annealing.

We characterized ZnO:Al films with various Al contents. Theoretically, the density of compensating acceptor-type v_Zn_″ increases with the concentration of donor-type Al_Zn_• because the formation energy of v_Zn_″ decreases with increases in the Fermi energy [4,5,6,7]. At the same time, the Al content of the films typically influences the optimum film growth temperature [11,12], and the extent of non-equilibrium in the as-deposited films increases with decreasing growth temperatures (*T*_g_). In this paper, we first describe the effects of *T*_g_ on the structural and electrical properties of ZnO:Al films with different Al contents. After optimizing *T*_g_ for each ZnO:Al film, we describe the variations in *n* and *μ* values during the post-annealing treatment at low and high *P*_O2_ and discuss the origin of the variations.

## 2. Results and Discussion

### 2.1. Properties of As-Deposited ZnO:Al Films

**Figure 1:**
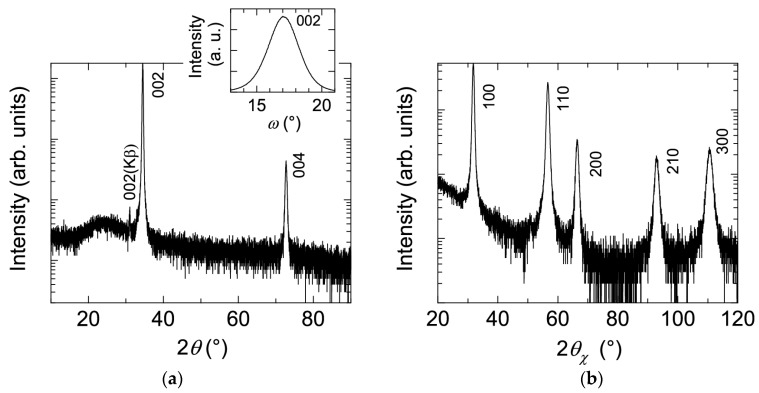
(**a**) *θ*–2*θ* scan (out-of-plane) with 002 *ω* scan; and (**b**) 2*θ_x_*–*ϕ* scan (in-plane) XRD patterns of a ZnO:Al (2.0 wt %) film.

**Figure 2:**
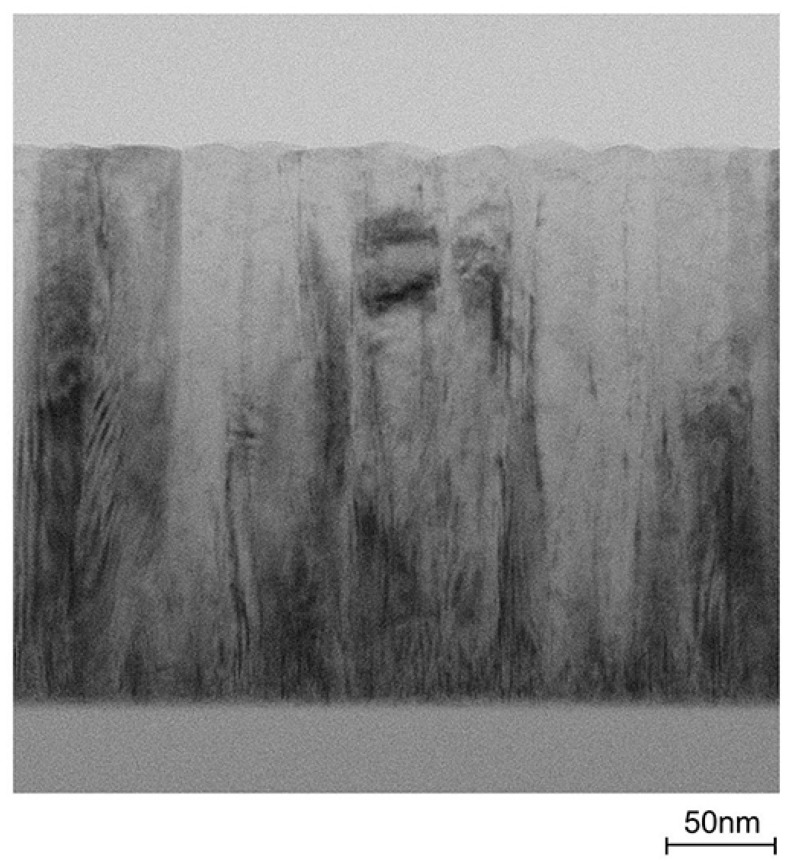
A cross-sectional TEM image of an as-deposited ZnO:Al (2.0 wt %) film.

Figure 1 shows (a) *θ*–2*θ* scan (out-of-plane) with 002 *ω* scan and (b) 2*θ_x_*–*ϕ* scan (in-plane) X-ray diffraction (XRD) patterns of a ~240-nm-thick ZnO:Al (2.0 wt %) film grown at 250 °C as an example. Only the diffraction peaks due to (002) and (004) planes were observed in the *θ*–2*θ* scan, whereas the diffraction peaks due to (100), (110), (200), (210), and (300) planes were observed. All of the prepared films with various Al contents and *T*_g_ exhibited preferred orientation of the *c*-axis normal to the substrate plane and did not contain any additional phases (e.g., ZnAl_2_O_4_ or Al_2_O_3_ phases). Because the solid solubility limit of Al in Al_2_O_3_-doped ZnO sintered body prepared at 1400 °C was reported to be ~0.3 at % [13], the ZnO:Al films fabricated in this study are metastable from a thermodynamic point of view, and a supersaturated solid solution occurs as a result of the highly non-equilibrium growth process. Figure 2 shows a cross-sectional transmission electron microscopy (TEM) image of the ZnO:Al (2.0 wt %) film. Abundant small columnar grains were present at the interface between the film and substrate, which had slightly tilted long-axis orientations normal to the substrate surface. For film thicknesses above 50 nm, the small grains became connected, and the deviation of each columnar grain decreased. Consequently, relatively large columnar grains with widths of 20–40 nm appeared when the film thickness exceeded 100 nm.

**Figure 3:**
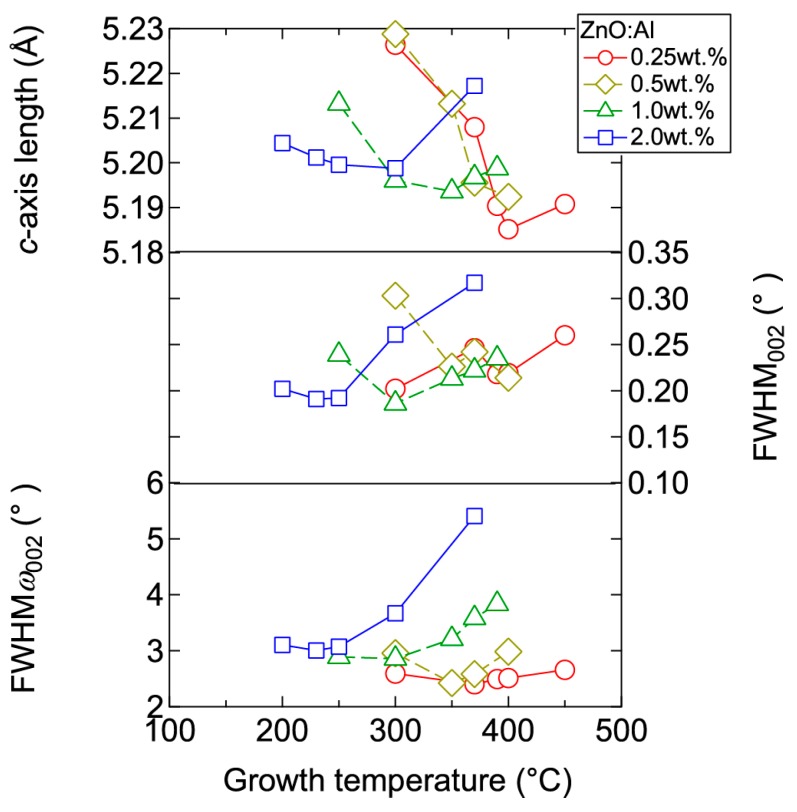
Plots of *c*-axis length, the full width at half maximum (FWHM) of the 002 diffraction peak (FWHM_002_), and the FWHM of rocking curves for the 002 diffraction peak (FWHM*ω*_002_) as a function of growth temperature for ZnO:Al (0.25, 0.5, 1.0, and 2.0 wt %) films.

Figure 3 displays plots of the *c*-axis length, the full width at half maximum (FWHM) of the 002 diffraction peak (FWHM_002_), and the FWHM of rocking curves for the 002 diffraction peak (FWHM*ω*_002_) as a function of *T*_g_ for the ~240-nm-thick ZnO:Al films. Here, FWHM_002_ reflects the out-of-plane crystallite size and the differences in *c*-axis length along the growth direction due to non-uniform micro-stresses, whereas FWHM*ω*_002_ reflects the *c*-axis tilt. For each ZnO:Al film with a given Al content, FWHM_002_ and FWHM*ω*_002_ decreased gradually with increasing *T*_g_ up to a certain optimal temperature (*T*_opt_), while these values increased above *T*_opt_. The values of *T*_opt_ were 250, 300, 350, and 370 °C for ZnO:Al films fabricated using ZnO ceramic targets containing 2.0, 1.0, 0.5, and 0.25 wt % Al_2_O_3_, respectively. *T*_opt_ decreases with increasing Al content. The variation of the FWHM values can be explained as follows: The diffusion length of adatoms and precursors at the growth surface increase with *T*_g_ up to *T*_opt_, thereby leading to greater crystallinity in the films. Conversely, further increases in *T*_g_ would significantly promote diffusion of the adatoms and precursors, leading to migration of ions in the bulk films, and desorption of Zn at the growth surface. Consequently, excess Al may degrade the film crystallinity by forming local atomic structures similar to a homologous phase ((ZnO)_m_Al_2_O_3_) [14,15]. The effect of inserting homologous phases in a ZnO film would be to increase the out-of-plane dimension of the film. Indeed, we observed an increase in the *c*-axis length at *T*_g_ above *T*_opt_ (Figure 3).

**Figure 4:**
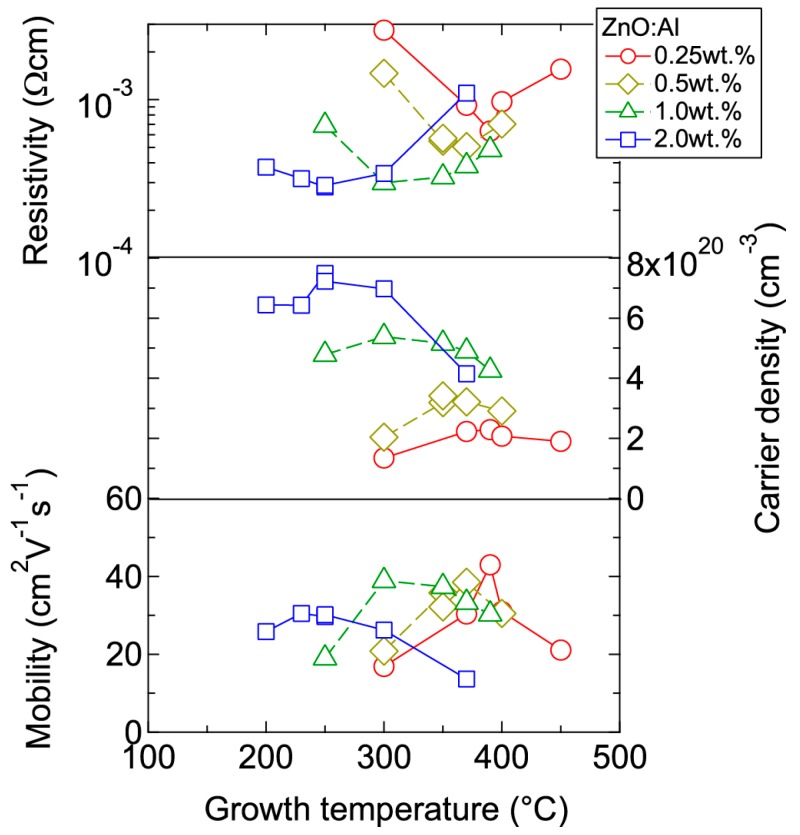
Plots of resistivity, carrier density, and mobility in ZnO:Al (0.25, 0.5, 1.0, and 2.0 wt %) films as a function of growth temperature.

**Figure 5:**
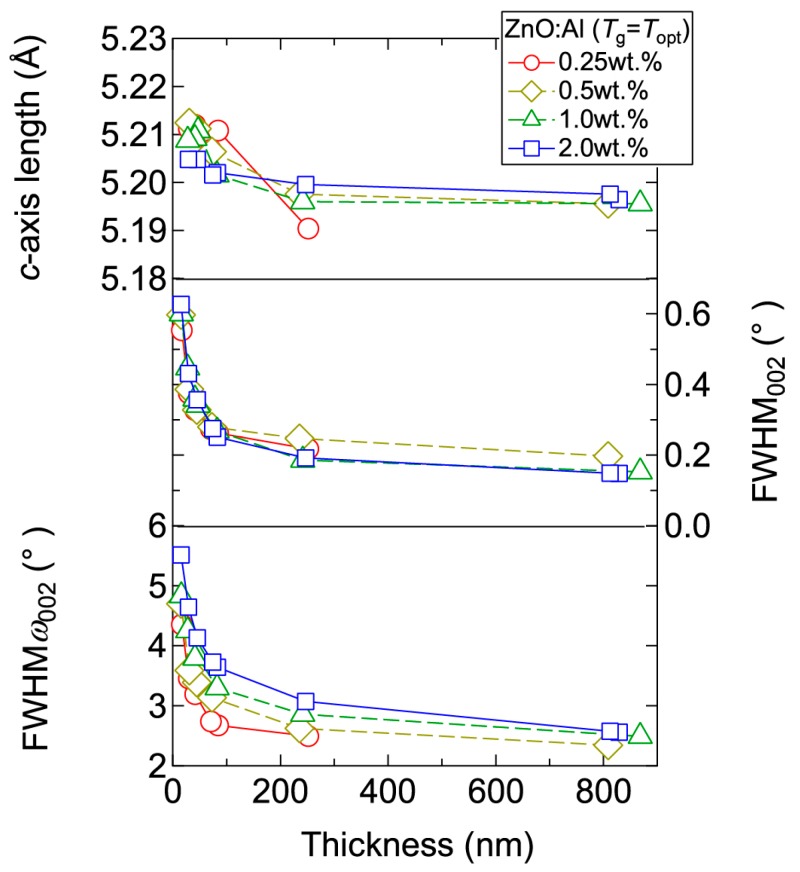
Plots of *c*-axis length, the FWHM of the 002 diffraction peak (FWHM_002_), and the FWHM of rocking curves for the 002 diffraction peak (FWHM*ω*_002_) as a function of thickness for ZnO:Al (0.25, 0.5, 1.0, and 2.0 wt %) films.

**Figure 6:**
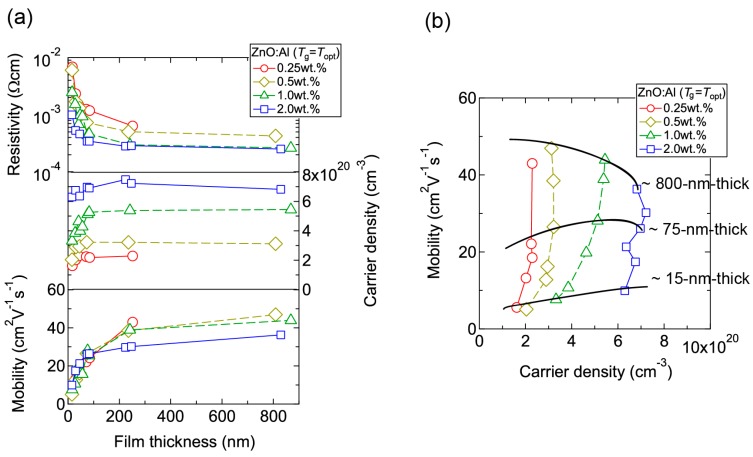
(**a**) Plots of resistivity, carrier density, and mobility in ZnO:Al (0.25, 0.5, 1.0, and 2.0 wt %) films as a function of film thickness; (**b**) Relationship between carrier density and mobility for the films.

Figure 4 displays the resistivity (*ρ*), *n*, and *μ* of the films plotted as a function of *T*_g_. With decreasing Al content, *n* monotonically decreased along with a gradual increase in *μ*. Consequently, *ρ* gradually increased with decreasing Al content. More importantly, in each ZnO:Al (0.25, 0.5, 1.0, and 2.0 wt %) film, both *n* and *μ* exhibited maximum values at the *T*_opt_ values indicated in Figure 3. The results suggest that imperfections of crystal are the cause of carrier trapping and scattering. This phenomenon is also observed in ZnO:Al films prepared at different film thicknesses. Figure 5 shows *c*-axis length, FWHM_002_, and FWHM*ω*_002_ as a function of film thickness for each ZnO:Al film deposited at *T*_opt_. Regardless of Al content, both FWHM_002_ and FWHM*ω*_002_ decreased rapidly with increasing film thickness up to 100–200 nm, and decreased gradually over 100–200 nm. Corresponding to the change in crystalline quality, both *n* and *μ* increased rapidly with increasing film thickness up to 100–200 nm, and increased gradually over 100–200 nm for each ZnO:Al (0.25, 0.5, 1.0, and 2.0 wt %), as shown in Figure 6a. Figure 6b plots a relationship between *n* and *μ*. In Figure 6b, thick solid lines are connected to the films having similar film thicknesses. In general, mobility of degenerated semiconductors decreases with increase in carrier density, since donor impurities work as scattering centers of free carriers. Indeed, measured *μ* for thick films with a thickness of ~800 nm decreased with an increase in Al content or *n*. However, for thin films with a thickness of less than 100 nm, mobility increased with carrier density. The behavior can be explained on the basis of scattering at the grain boundary (GB) rather than ionized impurities [16,17]. GB scattering is described by the Seto model, in which the mobility is dominated by thermionic emission across grain barriers, where impurities or other defects induce electron traps at GBs [16]. For films with very high *n* values, the depletion width formed at a GB is narrow, enabling tunneling through the barriers by free electrons. These results shown in Figure 3, Figure 4, Figure 5 and Figure 6 clearly indicate that imperfections in the crystal lattice and concentration of GB are the cause of carrier trapping and scattering. It should be noted that the values of *n*, *μ*, and *T*_opt_ obtained for each ZnO:Al are comparable to those previously reported for ZnO:Al films [11,12,18].

**Figure 7:**
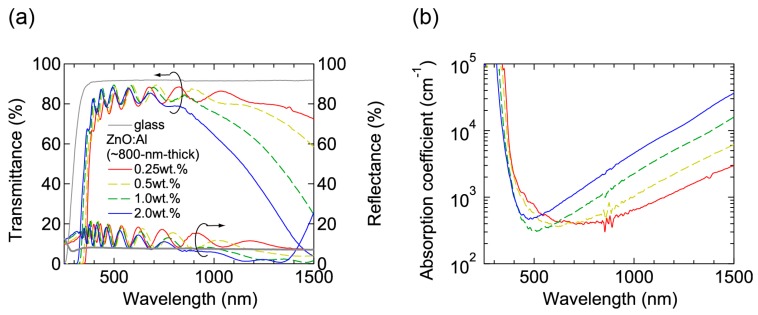
(**a**) Transmittance and reflectance; and (**b**) absorption coefficient spectra of the ~800-nm-thick ZnO:Al (0.25, 0.5, 1.0, and 2.0 wt %) films.

Figure 7 shows (a) transmittance and reflectance; and (b) absorption coefficient spectra of the ~800-nm-thick films grown at *T*_opt_. All the films were transparent in visible wavelength region and a decrease in transmittance in near-infrared wavelength region was observed due to free-carrier absorption. In the ultraviolet wavelength region, an onset wavelength, at which absorption began to increase, shifted to shorter wavelengths with increasing Al content due to Burstein-Moss shift.

### 2.2. Properties of ZnO:Al Films Post-Annealed at Low and High Oxygen Partial Pressures

**Figure 8:**
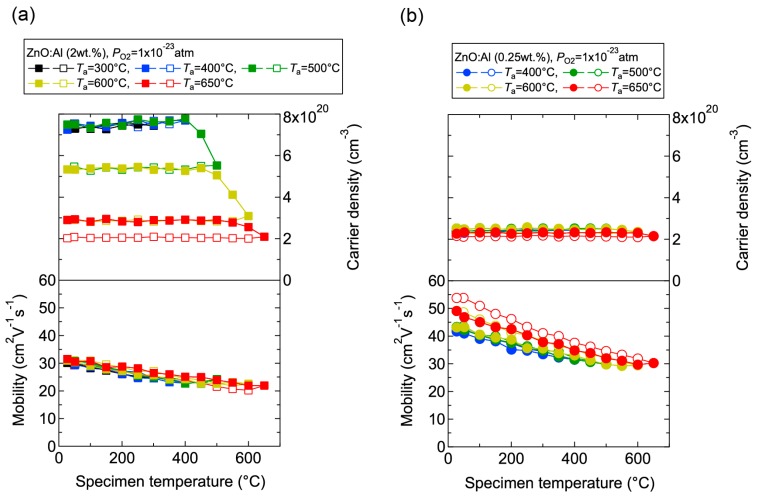
Changes in carrier density and mobility in (**a**) ZnO:Al (2.0 wt %) and (**b**) ZnO:Al (0.25 wt %) films as a function of specimen temperature during post-annealing treatments at a *P*_O2_ of 1 × 10^−23^ atm.

**Figure 9:**
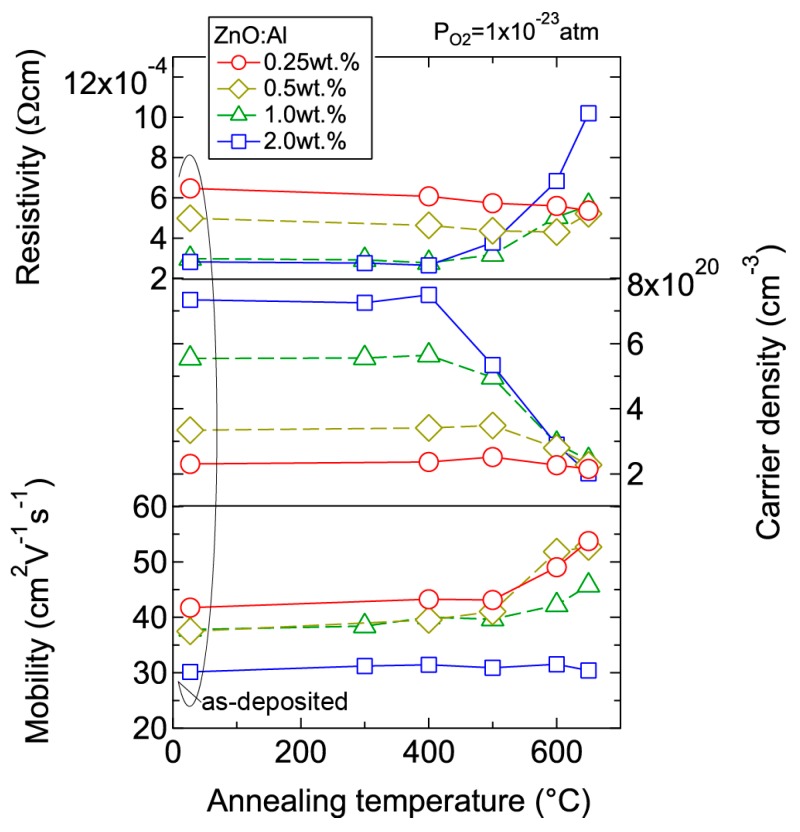
Room-temperature resistivity, carrier density, and mobility in ZnO:Al (0.25, 0.5, 1.0, and 2.0 wt %) films as a function of annealing temperature. Post-annealing was performed at a *P*_O2_ of 1 × 10^−23^ atm.

We investigated the effects of post-annealing treatments at low (1 × 10^−23^ atm) and high (1 × 10^−4^ atm) *P*_O2_ in ZnO:Al (0.25, 0.5, 1.0, 2.0 wt %) films grown at *T*_opt_. Figure 8 shows the changes in *n* and *μ* values for ZnO:Al (2.0 wt %) (Figure 8a) and ZnO:Al (0.25 wt %) (Figure 8b) films at a *P*_O2_ of 1 × 10^−23^ atm as a function of specimen temperature (*T*_s_) during post-annealing using a Hall measurement system. The measurement system is described in Section 3. As shown in Figure 8a, five post-annealing treatments were performed: (i) 50–300–50°C; (ii) 50–400–50 °C; (iii) 50–500–50 °C; (iv) 50–600–50 °C; and (v) 50–650–50 °C. Closed (open) symbols represent data measured during heating (cooling) in each post-annealing treatment. No large changes in *n* and *μ* were observed up to 400 °C, whereas a large decrease in *n* was observed with increasing *T*_s_ above 400 °C. The values of *n* during cooling in each annealing treatment were nearly identical to the *n* value at the maximum annealing temperature (*T*_a_). Furthermore, the samples also exhibited similar *n* values during heating in each subsequent annealing treatment up to temperatures less than the maximum *T*_a_ in the previous annealing treatment. These results clearly demonstrate that the equilibration time rapidly increases with decreasing *T*_s_. Therefore, the *n* values measured during the cooling process reflect quasi-equilibrium states and frozen defect densities at the maximum *T*_a_ of the annealing treatment. Similar changes in *n* values as a function of *T*_s_ were observed for the ZnO:Al (0.25 wt %) film, as shown in Figure 8b. However, the changes in *n* values were very small compared to those of the ZnO:Al (2 wt %) film. Figure 9 summarizes the changes in *ρ*, *n*, and *μ* values for the ZnO:Al (0.25, 0.5, 1.0, 2.0 wt %) films measured at room temperature after the post-annealing treatments at a *P*_O2_ of 1 × 10^−23^ atm. The data are plotted as a function of the maximum *T*_a_ in each annealing treatment. Interestingly, the overall behavior on heating was strongly dependent on the Al content in the films. For the ZnO:Al (0.25 wt %) film, *μ* increased from 42 to 54 whereas *n* did not change significantly with increasing *T*_a_. In contrast, for the ZnO:Al (2.0 wt %) film, *n* decreased rapidly at *T*_a_ above 400 °C, whereas *μ* did not vary at all, as also shown in Figure 8a,b. Furthermore, *n* values began to decrease at lower *T*_a_ values for ZnO:Al films with higher Al contents, resulting in *n* values after the high-*T*_a_ annealing treatments that were independent of the Al content: 5.0–5.3 × 10^20^ cm^−3^ for ZnO:Al (2.0 and 1.0 wt %) films (*T*_a_ = 500 °C), 2.8–2.9 × 10^20^ cm^−3^ for ZnO:Al (2.0, 1.0, and 0.5 wt %) films (*T*_a_ = 600 °C), and 2.0–2.4 × 10^20^ cm^−3^ for ZnO:Al (2.0, 1.0, 0.5, and 0.25 wt %) films (*T*_a_ = 650 °C).

**Figure 10:**
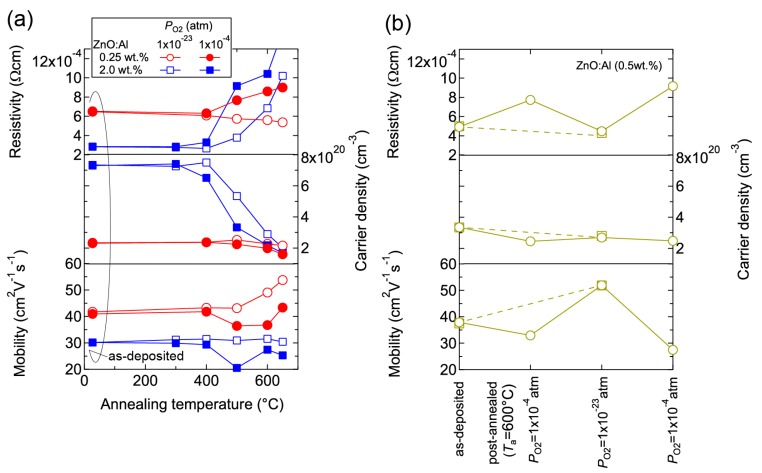
Room-temperature resistivity, carrier density, and mobility in (**a**) ZnO:Al (0.25 and 2.0 wt %) films plotted as a function of annealing temperature at *P*_O2_ values of 1 × 10^−23^ and 1 × 10^−4^ atm and (**b**) two ZnO:Al (0.5 wt %) films post-annealed at 600 °C under various *P*_O2_ values.

The observed changes in *n* and *μ* were dependent on both *T*_a_ and *P*_O2_. Figure 10 shows room temperature values of *ρ*, *n*, and *μ* for ZnO:Al (0.25 and 2.0 wt %) films plotted as a function of *T*_a_ at *P*_O2_ values of 1 × 10^−23^ and 1 × 10^–4^ atm (Figure 10a) and the ZnO:Al (0.5 wt %) films post-annealed at 600 °C at various *P*_O2_ values (Figure 10b). Figure 10a indicates that the *μ* values for the ZnO:Al (0.25 wt %) film increased only after post-annealing at low *P*_O2_. In addition, the *n* values for the ZnO:Al (0.25 and 2.0 wt %) films decreased significantly after annealing at a higher *P*_O2_. Figure 10b shows the results for two films that were post-annealed at 600 °C at different *P*_O2_ values. One film was annealed at a *P*_O2_ of 1 × 10^−23^ atm (dotted lines), while the other film was annealed at a *P*_O2_ of 1 × 10^−4^ atm, followed by an annealing treatment at a *P*_O2_ of 1 × 10^−23^ atm and a subsequent annealing treatment at a *P*_O2_ of 1 × 10^−4^ atm (solid lines). Both films exhibited similar transport properties after the annealing treatments at the same *P*_O2_ (1 × 10^−23^ or 1 × 10^−4^ atm), thereby reflecting their thermodynamic quasi-equilibrium states.

**Figure 11:**
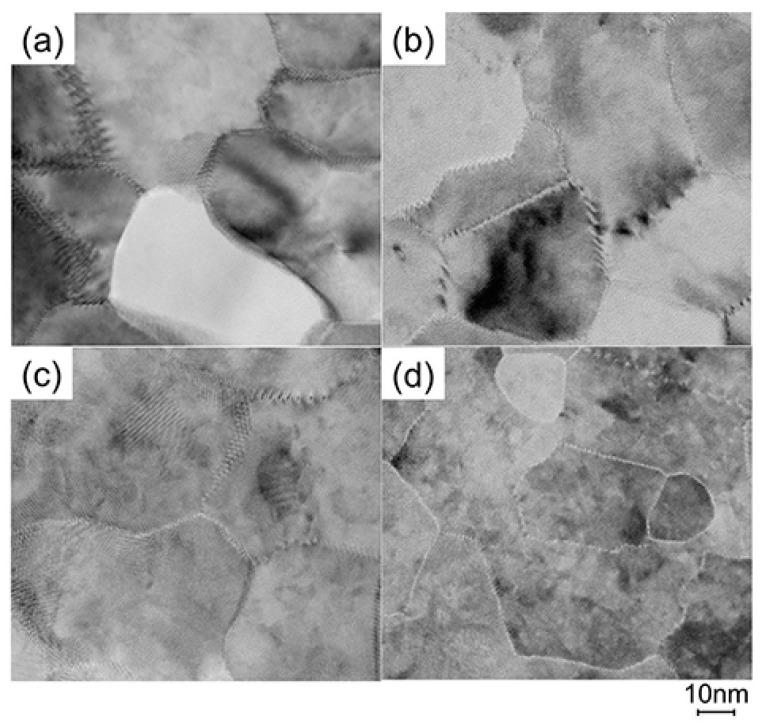
Plan-view TEM images of the as-deposited films of (**a**) ZnO:Al (0.25 wt %) and (**b**) ZnO:Al (2.0 wt %); and post-annealed films of (**c**) ZnO:Al (0.25 wt %) and (**d**) ZnO:Al (2.0 wt %) films at 650 °C at a *P*_O2_ of 1 × 10^−23^ atm.

**Figure 12:**
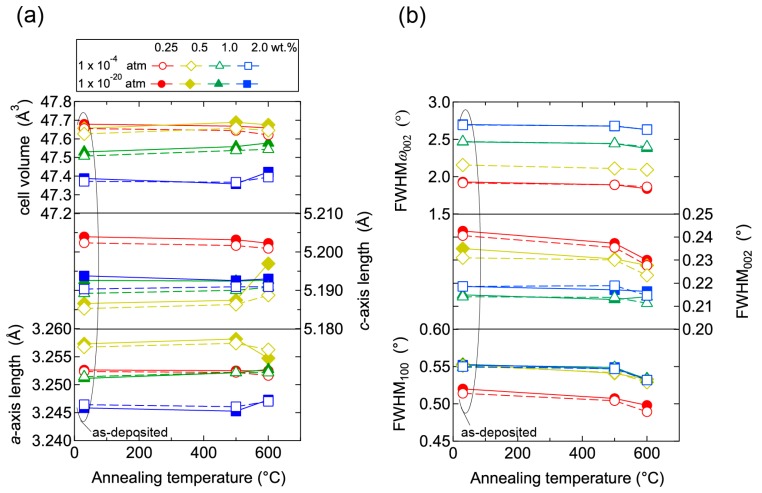
Plots of (**a**) *a*- and *c*-axis lengths and cell volumes as a function of annealing temperature, and (**b**) FWHM of rocking curves for 002 diffraction peaks (FWHM*ω*_002_) and FWHM of 002 and 100 diffraction peaks (FWHM_002_, FWHM_100_) as a function of annealing temperature for films post-annealed at a *P*_O2_ of 1 × 10^−20^ (**solid lines**) and of 1 × 10^−4^ (**dotted lines**) atm.

To elucidate the origin of the changes in *n* and *μ*, we characterized the structural properties of the films using TEM and XRD, and the chemical properties using X-ray photoelectron spectroscopy (XPS) and thermal desorption spectroscopy (TDS). Figure 11 displays plan-view TEM images of the as-deposited and post-annealed ZnO:Al (0.25 and 2.0 wt %) films at 650 °C. Most grain boundaries observed in the films were [001] tilt-type boundaries with a [001] rotation angle. This is because both films exhibited *c*-axis preferred orientations, as indicated by the FWHM*ω*_002_ of ca. 2°–3° (Figure 3). Figure 11a,b clearly shows that both of the as-deposited films contained strained regions that propagated toward the grain interior from the GBs. The strained regions are assumed to have been produced by unavoidable orientation mismatches between adjacent columns during crystal growth. After the post-annealing treatment, many of the boundaries appeared flat and located parallel to the *c*-axis at least within a thickness of the TEM foil for both films (Figure 11c,d). This feature may be attributed to grain growth that reduces the grain boundary area to achieving energetically favorable boundaries. Indeed, the FWHM_100_ slightly decreased with *T*_a_. Figure 12 shows the *a*- and *c*-axis lengths and cell volume (Figure 12a) along with FWHM*ω*_002_, FWHM_002_, and FWHM_100_ (Figure 12b) for the post-annealed films at *P*_O2_ values of 1 × 10^−20^ and 1 × 10^−4^ atm as a function of *T*_a_. These values were strongly dependent on the Al content as a result of the strong influence of *T*_g_ and thickness on the structural properties, as discussed in Section 2.1. Figure 12b shows that the FWHM_002_ and FWHM_100_ decreased with *T*_a_ above 500 °C, with the same changes observed at both low and high *P*_O2_. Furthermore, no significant differences in the other measured values were detected between the films post-annealed at low and high *P*_O2_. In contrast, the *μ* and *n* values shown in Figure 10 strongly depended on *P*_O2_. Therefore, the decrease in the strained region around the GBs and a slight increase in the in-plane crystallite size indicated in Figure 11 and Figure 12 were not responsible for the increase in *μ*. Additionally, the different *n* values produced by post-annealing at different *P*_O2_ values are not due to changes in the structural properties observed by TEM and XRD analysis.

**Figure 13:**
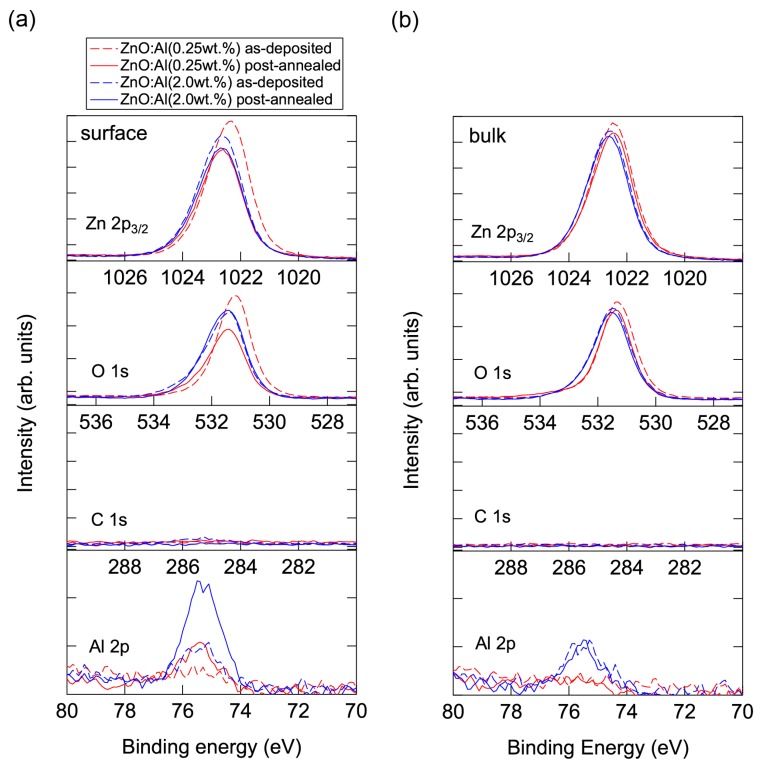
XPS spectra for (**a**) the surface regions and (**b**) the bulk regions of the as-deposited and post-annealed ZnO:Al (0.25 and 2.0 wt %) films at a *P*_O2_ of 1 × 10^−23^ atm.

**Figure 14:**
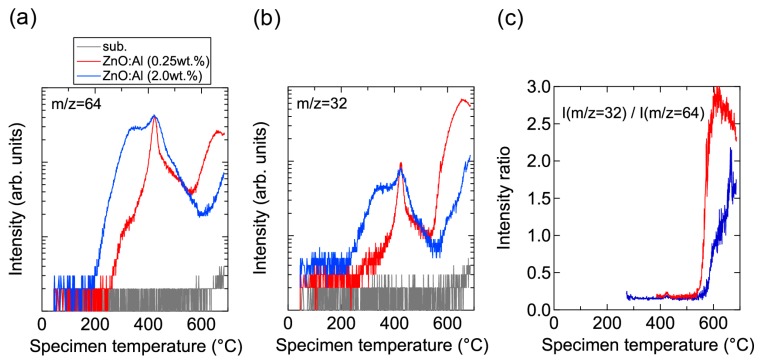
TDS spectra of desorption species with masses (m/z) of (**a**) 64 and (**b**) 32 from ZnO:Al (0.25 wt %) and ZnO:Al (2.0 wt %) films grown at optimized temperatures; (**c**) The intensity ratio of m/z = 32 to m/z = 64.

Conversely, high-temperature annealing at low *P*_O2_ is likely to change the chemical properties of the films. Figure 13 shows XPS spectra for the surface regions (Figure 13a) and bulk regions (Figure 13b) of the as-deposited and post-annealed ZnO:Al films. Figure 13a indicates that the Al contents in the surface regions were significantly higher than in the bulk films for both post-annealed ZnO:Al (0.25 and 2.0 wt %) films. The concentrations of Al, Zn, and O (*C*_Al_ (at %), *C*_Zn_ (at %), and *C*_O_ (at %)) estimated from the XPS spectra are summarized in Table 1. The results suggest that minor decomposition of the ZnO layer occurs in the surface region during high-temperature annealing at low *P*_O2_. This is also supported by the TDS spectra for the ZnO:Al films. Figure 14 shows the TDS spectra of desorption species from the ZnO:Al (0.25 wt %) and ZnO:Al (2.0 wt %) films grown at *T*_opt_. We monitored the mass (m/z) of (a) 64; (b) 32; and (c) the ratio between the two. Here, an m/z value of 64 includes only Zn^+^, whereas an m/z of 32 includes both Zn^2+^ and O_2_^+^. The behavior of the m/z = 32 signals were similar to those of the m/z = 64 signals with a constant ratio, especially for *T*_s_ below 530 °C, thereby indicating that most of the m/z = 32 signals below 530 °C reflect the desorption of Zn rather than O_2_. In contrast, at temperatures above 530 °C, the m/z = 32 signals sharply increased with *T*_s_ and exceeded the level of the m/z = 64 signals, thereby indicating that O_2_ was also desorbed. Hence, decomposition of the films began at *T*_s_ values above 500 °C in a high vacuum (i.e., <1 × 10^−7^ Pa).

Prior to the onset of decomposition, defects within grains are likely to change during the post-annealing treatment at high temperatures and at low *P*_O2_. As observed in Figure 14a, Zn became desorbed from the surface of the films at temperatures above ~200 °C. Furthermore, a larger amount of Zn was desorbed from the ZnO:Al (2.0 wt %) film compared to the ZnO:Al (0.25 wt %) film. Although the desorption behavior was complex, the signals exhibited peaks at ~350 °C and ~430 °C with a shoulder at ~470 °C in both films. These behaviors are considered to reflect changes in Zn-related defects in the ZnO:Al films. During post-annealing, Zn interstitials diffused from the bulk layers, especially along GBs, and became desorbed from the film surface. A potential source of the Zn interstitial is excess Zn inside the films. Another candidate is Zn interstitials that were generated by the removal of Zn from the ZnO lattice along with the generation of Zn vacancy during the post-annealing treatment, with acceptor-type Zn vacancy remaining inside the grains. First-principle density functional theory suggests that acceptor-type v_Zn_″ defects are formed and that carrier compensation occurs in ZnO:Al with increasing *P*_O2_ [4,5,6,7]. The density of v_Zn_″ increases with Al content in ZnO:Al because the formation energy of v_Zn_″ decreases with increases in the Fermi energy of ZnO. In addition, Zn_i_•• can diffuse even through the bulk lattice due to a low migration barrier [7]. Based on the combination of these theoretical suggestions, a decrease in *n* at temperatures above 400 °C—especially for heavily Al-doped ZnO films at high *P*_O2_ (Figure 9 and Figure 10), desorption of Zn at these temperatures (~430 °C) under a high vacuum (i.e., <1 × 10^−7^ Pa) (Figure 14a), and no presence of Zn vapor during the post-annealing processes under the constant *P*_O2_—Zn interstitials and Zn vacancies are thought to be created in the ZnO lattice during post-annealing at temperatures above 400 °C. Therefore, Zn desorption from the films and carrier compensation by acceptor-type v_Zn_″ occurred simultaneously. In contrast, desorption of Zn below 400 °C had no influence on the *n* values, as observed in Figure 8, Figure 9, and Figure 14. The results suggest that excess Zn that does not contribute to carrier generation is present within the as-deposited films. Although the formation energy of a Zn_i_•• is high, and thus high densities of Zn_i_•• are not expected in ZnO at equilibrium conditions [4,6,7], a large amount of Zn may be introduced into the films grown using a non-equilibrium growth method, especially when grown under Zn-rich conditions at low temperatures. Indeed, several papers have also reported the detection of Zn desorption from sputtered ZnO films in TDS spectra for films grown under low O_2_/Ar flow ratios [19,20,21,22]. It should be noted that Zn desorption decreases with increasing O_2_ desorption at high O_2_/Ar flow ratios [22]. In addition, we observed a monotonic decrease in *n* with increases in the O_2_/(Ar + O_2_) flow ratio: 1.7 × 10^20^, 3.1 × 10^20^, 5.1 × 10^20^, and 5.4 × 10^20^ cm^−3^ for ZnO:Al films grown at a ratio of 1%, compares with 1.4 × 10^20^, 2.3 × 10^20^, 4.3 × 10^20^, and 3.8 × 10^20^ cm^−3^ for ZnO:Al films grown at a ratio of 2% with Al_2_O_3_ contents of 0.25, 0.5, 1.0, and 2.0 wt %, respectively. These results suggest that thin-film growth during sputtering without the introduction of O_2_ produces Zn–rich conditions. Such conditions will prevent the generation of large amounts of v_Zn_″ during growth and heavy carrier compensation by the v_Zn_″.

The large increase in *μ* after low-*P*_O2_ annealing for ZnO:Al films with low Al contents can be explained by a reduction in scattering centers at GBs. As described in Section 2.1, *μ* for the ZnO:Al films is influenced by GB scattering in addition to scattering from ionized impurities. Figure 10b illustrates that *μ* increased after low-*P*_O2_ annealing, whereas *μ* decreased after high-*P*_O2_ annealing. As shown in Figure 12, no notable change in the structural properties was observed between the films post-annealed at low and high *P*_O2_. In contrast, we detected a change in the chemical states of the film surfaces by XPS and TDS measurements in films post-annealed at low *P*_O2_. These results suggest that point defects at GBs that act as scattering centers are reduced by low-*P*_O2_ annealing. Conversely, we did not observe any increase in *μ* in a post-annealed ZnO:Al (2.0 wt %) film with a similar *n* value, likely due to an increase in the compensation ratio by the generation of large amount of v_Zn_″, as discussed above.

Based on these results, carrier compensation occurs by the generation of v_Zn_″acceptor-type defects when sputtered ZnO:Al films are post-annealed at high temperatures. The compensation behavior differs with Al content. For high-Al ZnO:Al films (i.e., 2.0 wt %), *n* starts to decrease at ~400 °C even at a low *P*_O2_ of 1 × 10^−23^ atm, whereas in low-Al films (i.e., 0.25 wt %), *n* starts to decrease at ~600 °C. Furthermore, the amount of desorbed Zn and the amount of acceptor-type defects estimated from the changes in *n* values increase with Al content. These results are in good agreement with theoretical predictions [6]. Practical applications require higher *n* and *μ* values. Low-*P*_O2_ annealing is effective in increasing *μ* values, likely due to reduction in the amount of point defects at GBs that act as scattering centers. However, at present, *n* values are small compared to the Al content and a portion of the free carriers is likely to be trapped by crystal imperfections, as described in Section 2.1. In this case, controlling the presence of tilt-type GBs in Zn-rich conditions will be important [23]. Additionally, Zn vacancies and other related defects have been detected in as-deposited ZnO films by positron annihilation [24,25]. Self-compensation by the production of v_Zn_″ has also been experimentally demonstrated for as-deposited Ga-doped ZnO films [10]. Therefore, passivation of the existing v_Zn_″ defects by a post-annealing treatment can be useful. For example, post-annealing in a Zn atmosphere is a candidate method for reducing v_Zn_″ concentrations. Experimental studies have also reported that post-annealing treatments in a Zn atmosphere improve both *n* and *μ* values [26,27]. Also, hydrogen has been proposed to passivate acceptor-type cation vacancies [28]. Other experiments have also found that annealing treatments in forming gas (5%–10% H_2_/balance Ar or N_2_) [10,27,29] and hydrogen diffusion from SiN_x_:H or a-Si:H layers [30,31] can improve both *n* and *μ* values.

## 3. Materials and Methods

ZnO:Al thin films were grown on heated alkali-free glass (Corning #1737) and SiO_2_-coated Si using a radio frequency magnetron sputtering system. Sintered ZnO ceramic targets containing 0.25, 0.5, 1.0, and 2.0 wt % Al_2_O_3_ powder were used as source materials and the radio frequency power density during sputtering was 4.4 W·cm^−2^. The distance between the target and the substrates (three glass and one SiO_2_-coated Si of 50 × 50 mm in size) was 100 mm and the base pressure in the deposition chamber prior to deposition was ~2 × 10^−5^ Pa. High-purity Ar gas was introduced at a gas flow rate of ~50 sccm and the deposition pressure was 0.2 Pa. No O_2_ gas was introduced to the deposition chamber. The film thicknesses were varied from 15 to 800 nm. The growth temperatures for ZnO:Al (0.25, 0.5, 1.0, and 2.0 wt %) films were 200–370, 250–390, 300–400, and 300–450 °C, respectively. After deposition, some films were post-annealed in N_2_ at a constant *P*_O2_ using an oxygen pump [32,33]. These films were grown at 370, 350, 300, and 250 °C for ~240-nm-thick ZnO:Al (0.25, 0.5, 1.0, and 2.0 wt %) films, respectively.

**Figure 15:**
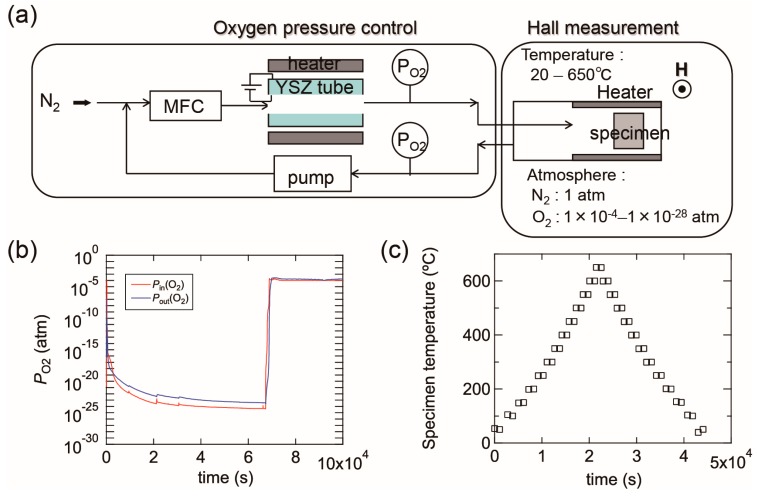
(**a**) A schematic image of our experimental setup composed of an oxygen pump and a Hall measurement system; (**b**) example diagram of *P*_O2_ variations as a function of time. The *P*_O2_ was controlled by changing the electric field between the inner and outer electrodes of a solid electrolyte tube made of yttria-stabilized zirconia (YSZ); (**c**) temperatures during (*ρ*, *n*, *μ*) measurements as a function of time. In this measurement, the electrical properties were measured every 50 °C during heating from 50 to 650 °C and subsequent cooling from 650 to 50 °C.

Figure 15a shows a schematic image of our experimental setup, which contains an oxygen pump and a Hall measurement system. The oxygen pump is composed of a solid electrolyte tube made of yttria-stabilized zirconia (YSZ). Platinum electrodes are attached to both its outer and inner surfaces and the YSZ tube is heated up to 700 °C. When N_2_ gas flows into the tube, the residual O_2_ molecules in the N_2_ gas are ionized at the surface of the inner electrode. The oxygen ions are then moved to the outer electrode by an electric field between the two electrodes, which results in the oxygen molecules being swept away outside the tube. This allows the *P*_O2_ of the N_2_ gas to be reduced during the flow through the tube. The *P*_O2_ can be controlled within the range of ~1 × 10^−4^ to ~1 × 10^−28^ atm by adjusting the electric field between the two electrodes. The oxygen pump is connected to the Hall measurement system and *P*_O2_ is monitored using oxygen sensors located at the inlet and outlet of the oxygen pump. Figure 15b shows an example of *P*_O2_ variations in the experimental setup in which *P*_O2_ gradually decreased down to less than 1 × 10^−23^ atm and then *P*_O2_ was immediately increased to 1 × 10^−4^ atm by changing the electric field. The *ρ*, *n*, and *μ* values of the films were obtained in a van der Pauw configuration. The Hall measurement system incorporates a heating system and the electrical properties can be measured at high temperatures of up to 650 °C in N_2_. The *T*_s_ values were monitored using a thermocouple located just behind the substrate. Most of the specimens on glass with a size of ~10 × 10 mm were post-annealed during the (*ρ*, *n*, *μ*) measurements, however some larger specimens on glass of 50 × 50 mm in size were post-annealed in a quartz furnace to characterize their structural properties using XRD. Figure 15c displays a plot of temperature before and after each (*ρ*, *n*, *μ*) measurement as a function of time. Electrical properties were measured every 50 °C while heating from 50 to 650 °C and were subsequently cooled from 650 to 50 °C with a ramping and cooling rate of ~10 K·min^−1^. The average time needed to achieve a constant temperature for the (*ρ*, *n*, *μ*) measurements was approximately 30 min, therefore, the entire measurement sequence was performed over 12 h. It should be noted that these annealing times are not sufficient to produce equilibrium states at the annealing temperatures used, and that the equilibration time will decrease with increases in *T*_s_.

**Figure 16:**
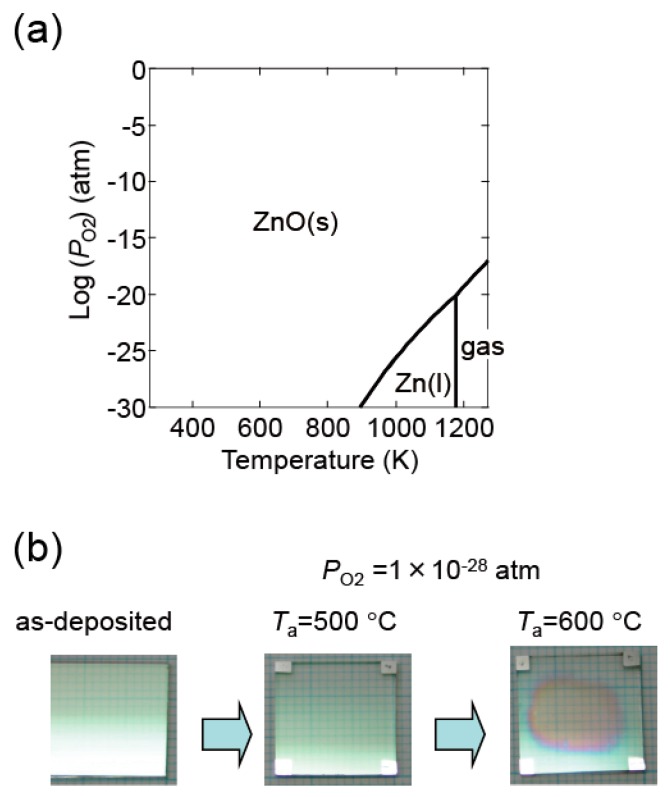
(**a**) Diagram indicating the conditions of thermodynamic equilibrium between ZnO and Zn + 1/2O_2_ for pure ZnO; (**b**) images of a ZnO:Al (0.25 wt %) film before and after post-annealing at 500 and 600 °C at a *P*_O2_ of 1 × 10^−28^ atm using the Hall measurement system.

Figure 16a shows a thermodynamic equilibrium diagram between ZnO and Zn + 1/2O_2_ for pure ZnO. ZnO decomposes at high temperatures and low *P*_O2_. In our experiments, ZnO:Al film decomposition was observed in certain conditions. Figure 16b contains an image of a ZnO:Al (0.25 wt %) film post-annealed at 600 °C at a *P*_O2_ of 1 × 10^−28^ atm using the Hall measurement system. Decomposition occurred in the center area of the film along with a reduction in thickness. The (*ρ*, *n*, *μ*) measurements described in this study were performed with post-annealing up to 650 °C at a *P*_O2_ of 1 × 10^−4^ and of 1 × 10^−23^ atm. At these conditions, we observed no change in color as a result of the reduction in thickness.

The chemical properties and composition of the films were analyzed by XPS using a monochromatic Al-K*α* X-ray source. To avoid carbon contamination at the surface, Ar^+^ ion etching was lightly performed until the carbon signal disappeared prior to the XPS measurements on the film surface regions. Conversely, XPS measurements on the bulk film regions were performed after extended Ar^+^ ion etching. The crystal structures were analyzed by XRD and TEM. *θ*–2*θ* scan (out-of-plane) and 2*θ_x_*–*ϕ* scan (in-plane) XRD measurements using Cu-K*α* radiation were performed. TDS was used to evaluate gas desorption during post-annealing. The base pressure of the TDS system was less than 2 × 10^−8^ Pa. For TDS measurements, the films were characterized on SiO_2_-coated Si with a size of 10 × 10 mm with a constant specimen heating rate of ~16 °C·min^−1^ using an infrared lamp. The annealing temperature was monitored using a thermocouple in contact with the specimen. The desorbed gases from the specimens were identified using a quadrupole mass spectrometer.

## 4. Conclusions

Carrier compensation effects induced by post-annealing treatments under low and high *P*_O2_ were investigated in ZnO:Al films. The ZnO:Al films were deposited by sputtering ZnO:Al ceramic targets (with 0.25, 0.5, 1.0, and 2.0 wt % Al_2_O_3_) under optimized growth conditions. The as-deposited films were post-annealed in N_2_ at a *P*_O2_ of 1 × 10^−23^ or 1 × 10^−4^ atm, controlled by an oxygen pump. The variations of the *n* and *μ* values during the post-annealing treatment were evaluated using a Hall measurement system. Both values were found to strongly depend on the Al content and *P*_O2_. For the ZnO:Al (0.25 wt %) film, the *μ* value increased from 42 to 54 cm^2^·V^−1^·s^−1^ whereas *n* exhibited similar values (2.3–2.2 × 10^20^ cm^−3^) after post-annealing at 650 °C at a *P*_O2_ of 1 × 10^−23^ atm. In contrast, the ZnO:Al (2.0 wt %) film exhibited *n* values that significantly decreased from 7.3 × 10^20^ to 2.0 × 10^20^ cm^−3^ whereas *μ* exhibited similar values (~30 cm^2^·V^−1^·s^−1^) after the same post-annealing treatment. Furthermore, the decrease in *n* values began at a lower *T*_a_ for ZnO:Al films with higher Al contents and this temperature further decreased with increasing *P*_O2_. Based on an analysis of the Hall, XRD, TEM, TDS, and XPS data, we concluded the following. (i) Post-annealing at temperatures above ~200 °C promotes desorption of Zn that was introduced into the films under non-equilibrium growth and Zn-rich conditions. These growth conditions likely suppress the generation of Zn vacancies in the lattice during growth. In addition, the Zn desorbed at temperatures below 400 °C does not contribute to carrier generation in the as-deposited films; (ii) Post-annealing at temperatures above 400 °C generates acceptor-type Zn vacancies in the lattice along with desorption of Zn from the films, which results in carrier compensation. In addition, the defect density increases with Al content and *P*_O2_; (iii) High-temperature (i.e., >500 °C) annealing at low *P*_O2_ (i.e., 1 × 10^−23^ atm) is effective in increasing *μ*, likely due to the reduction in point defects at GBs that act as carrier scattering centers.

## Figures and Tables

**Table 1:** The concentrations of Al, Zn, and O (*C*_Al_ (at %), *C*_Zn_ (at %), and *C*_O_ (at %)) estimated from the XPS spectra for the surface and bulk regions of the as-deposited and post-annealed ZnO:Al (0.25 and 2.0 wt %) films at a *P*_O2_ of 1 × 10^−23^ atm.

**Surface**	**ZnO:Al (0.25 wt %)**		**ZnO:Al (2.0 wt %)**	
	**As-Deposited**	**Post-Annealed**	**As-Deposited**	**Post-Annealed**
*C*_Al_	0.69	3.15	2.04	5.94
*C*_Zn_	48.18	48.44	46.10	41.52
*C*_O_	51.13	48.41	51.86	52.54
**Bulk**	**ZnO:Al (0.25 wt %)**		**ZnO:Al (2.0 wt %)**	
	**As-Deposited**	**Post-Annealed**	**As-Deposited**	**Post-Annealed**
*C*_Al_	0.46	0.65	2.14	2.60
*C*_Zn_	48.52	47.85	46.03	45.82
*C*_O_	51.02	51.50	51.83	51.58
